# An Algorithm (LaD) for Monitoring Childbirth in Settings Where Tracking All Parameters in the World Health Organization Partograph Is Not Feasible: Design and Expert Validation

**DOI:** 10.2196/17056

**Published:** 2021-05-27

**Authors:** Michael S Balikuddembe, Peter K Wakholi, Nazarius M Tumwesigye, Thorkild Tylleskar

**Affiliations:** 1 Center for International Health University of Bergen Bergen Norway; 2 Division of Maternal and Foetal Medicine Mulago Specialised Women and Newborn Hospital Mulago Hospital Kampala Uganda; 3 School of Computing and Information Technology Makerere University Kampala Kampala Uganda; 4 Department of Epidemiology and Biostatistics Makerere University School of Public Health Kampala Uganda

**Keywords:** algorithm, software validation, childbirth monitoring, WHO partograph

## Abstract

**Background:**

After determining the key childbirth monitoring items from experts, we designed an algorithm (LaD) to represent the experts’ suggestions and validated it. In this paper we describe an abridged algorithm for labor and delivery management and use theoretical case to compare its performance with human childbirth experts.

**Objective:**

The objective of this study was to describe the LaD algorithm, its development, and its validation. In addition, in the validation phase we wanted to assess if the algorithm was inferior, equivalent, or superior to human experts in recommending the necessary clinical actions during childbirth decision making.

**Methods:**

The LaD algorithm encompasses the tracking of 6 of the 12 childbirth parameters monitored using the World Health Organization (WHO) partograph. It has recommendations on how to manage a patient when parameters are outside the normal ranges. We validated the algorithm with purposively selected experts selecting actions for a stratified sample of patient case scenarios. The experts’ selections were compared to obtain pairwise sensitivity and false-positive rates (FPRs) between them and the algorithm.

**Results:**

The mean weighted pairwise sensitivity among experts was 68.2% (SD 6.95; 95% CI 59.6-76.8), whereas that between experts and the LaD algorithm was 69.4% (SD 17.95; 95% CI 47.1-91.7). The pairwise FPR among the experts ranged from 12% to 33% with a mean of 23.9% (SD 9.14; 95% CI 12.6-35.2), whereas that between experts and the algorithm ranged from 18% to 43% (mean 26.3%; SD 10.4; 95% CI 13.3-39.3). The was a correlation (mean 0.67 [SD 0.06]) in the actions selected by the expert pairs for the different patient cases with a reliability coefficient (α) of .91.

**Conclusions:**

The LaD algorithm was more sensitive, but had a higher FPR than the childbirth experts, although the differences were not statistically significant. An electronic tool for childbirth monitoring with fewer WHO-recommended parameters may not be inferior to human experts in labor and delivery clinical decision support.

## Introduction

From the late 20th century, there were concerted efforts to improve pregnancy outcomes, with the World Health Organization (WHO) partograph being the main labor monitoring tool used globally [1-3]. Increasing and easing of childbirth monitoring have been at the forefront of strategies for better maternal and newborn outcomes [4-6]. A spiraling increase in the number of caesarean sections due to prolonged labor led to research that challenged the cervical dilatation rates in the partograph [7-9]. Doubt arose on the validity of the partograph and intrapartum guidelines with calls for their re-evaluation [5,6,10]. Calls for more evidence-based care at birth led to increased research for more practical labor monitoring guidelines and tools [7,11-13].

In 2015, the American college of Obstetricians and the Society for Maternal-Fetal Medicine issued new guidelines on labor monitoring [14]. Later, the WHO released new recommendations on partograph use including calls for more research on the most appropriate paper-based or electronic tool to aid childbirth decision making [12]. Before any electronic decision support can be developed, an algorithm is needed outlining which decisions to take at each potential situation along the birth of a child. The algorithm is also preceded by a decision on which input variables to use is needed. Among the problems with the WHO partograph was a large number of variables to register and it was regarded as labor intensive and unpractical for low-resource settings [4,15]. We studied the labor monitoring tool expectations of childbirth experts in Africa to generate consensus on the most important parameters to monitor during birth in low-resource settings [16,17]. The findings included a reduction in the WHO-modified partograph items and several suggestions on changing the frequency of monitoring the labor items. The experts also expressed a need to adopt the recommendation for raising the starting point of the partograph from 4 cm of cervical dilatation.

In this paper, we describe the labor and delivery (LaD) algorithm, its development, and validation. In the validation we wanted to know if the algorithm is inferior, equivalent, or superior to human experts in recommending the necessary clinical actions during childbirth decision making.

## Methods

### Overview

We used the maternity experts’ recommendations and literature findings to develop an alternative algorithm for labor and delivery monitoring (the LaD algorithm). We conducted a preliminary validation of its logic before fully implementing it. Because of lack of a gold standard against which to compare the logic, we compared it against opinions of experts in childbirth monitoring. Comparison of results from medical devices against experts is increasingly seen as the better alternative when no gold standard exists and decisions are highly dependent on opinions or anecdotal evidence [18-21].

### Development of the LaD Algorithm

From our earlier studies [16,17], the key parameters to monitor in childbirth were the fetal heart rate, amniotic fluid, cervical dilatation, uterine contractions, maternal blood pressure, and pulse rate. The suggested monitoring intervals ranged from 30 minutes to 4 hours. These are 6 of the 12 parameters in the WHO-modified partograph [22]. We used these recommendations and literature on the progress and outcomes of monitoring various childbirth items to generate a parameter list and monitoring intervals to include in the algorithm. Our main adjustment to the experts’ suggestions was replacing the maternal pulse with second-stage tracking of the fetal station (a surrogate for fetal descent).

**Figure 1:**
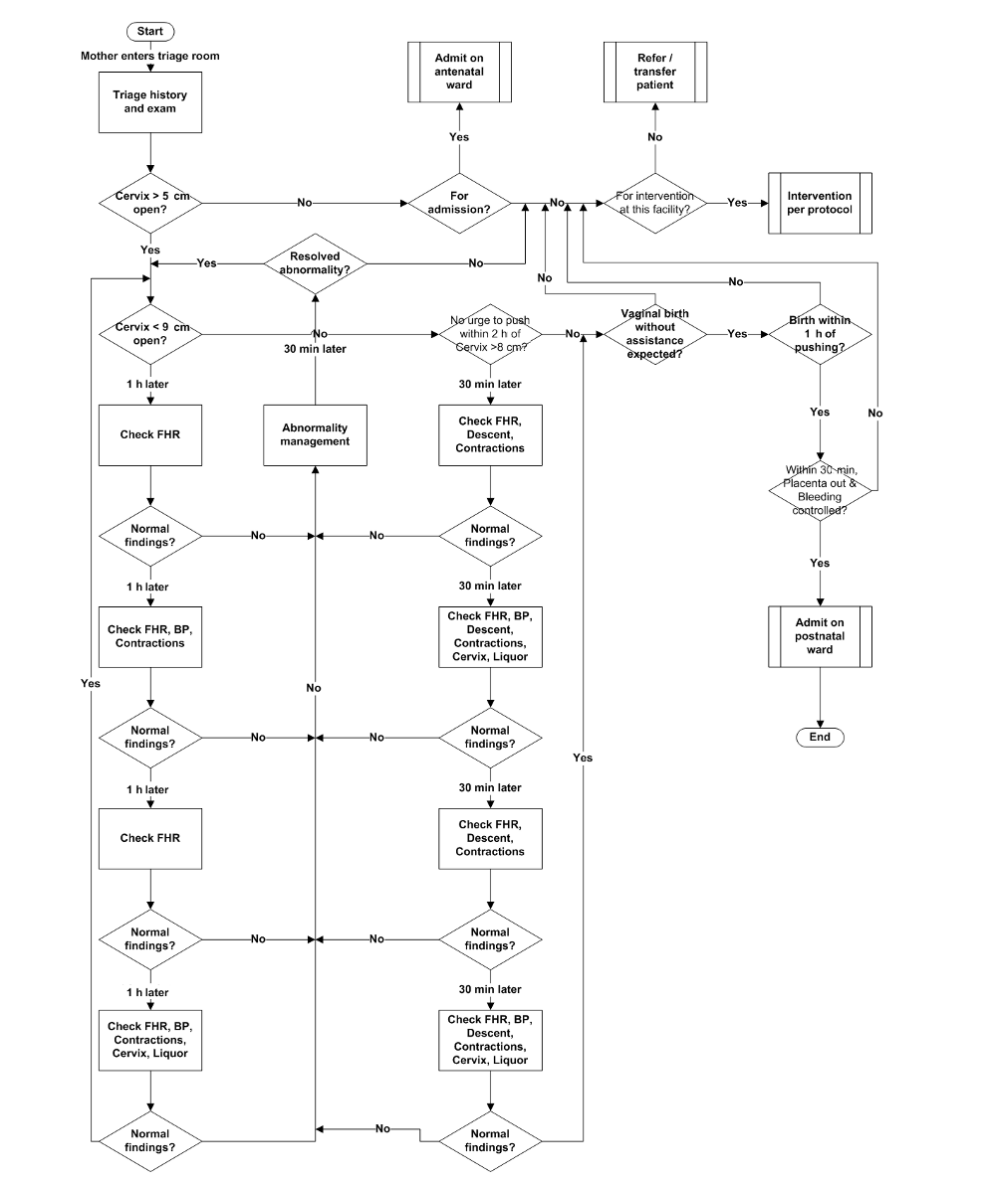
The LaD algorithm for monitoring labor and delivery.

We used our acumen on labor progress and its monitoring process to draw the LaD algorithm using the Microsoft Visio 2013. It was revised to the layout shown in Figure 1. It shows the parameters to monitor at evidence-based time intervals.

For the algorithm to run on a computing device, we translated it into a recursive (ie, a problem is divided into subproblems of the same type. The solution to the problem is devised by combining the solutions obtained from the simpler parts of the problem) logic with 1152 possible patient scenarios and key decision support actions. Any abnormality in labor monitoring parameters is independently managed (as per local guidelines) and the final labor management decision is based on the success or failure in managing the subabnormalities. It is this logic that we validated with another group of childbirth experts.

### Validation of the LaD Algorithm

Between January and February 2019, 5 purposively selected childbirth care experts (E1, E2, E3, E4, and E5) independently answered a survey questionnaire covering 6 patient case scenarios (P1, P2, P3, P4, P5, and P6). The 5 experts had a mean experience of 17 years (SD 5.8 years) in medical practice and an obstetric career length ranging from 5 to 17 years (mean 10.6 years [SD 5.1 years]). Most worked in a teaching hospital, with their highest education level ranging from a master’s degree to a Doctor of Philosophy (Table 1).

**Table 1:** Summary characteristics of experts who participated in validation.

Characteristics	Expert 1	Expert 2	Expert 3	Expert 4	Expert 5	Mean (SD)
Experience as a doctor (years)	12	16	11	23	23	17 (5.8)
Experience as an obstetrician (years)	6	11	5	14	17	10.6 (5.1)
Number of times expert selected actions in the 5 scenarios (maximum 80)	36	34	44	35	46	39 (5.6)
Highest level of medical education	Master’s degree	Master’s degree	PhD candidate	Master’s degree	PhD	—
Primary workplace	Military hospital	Medical school	Medical school	National hospital	Medical school	—

The case scenarios were taken from childbirth scenarios in the algorithm using stratified sampling. The cases were stratified using the amniotic fluid status into 3 strata: membranes intact, amniotic fluid clear, and amniotic fluid opaque or foul smelling. An online random number generator [23] was used to randomly select 2 cases from each stratum. The questionnaire had 15 labor-related conditions and 22 actions to consider. Each expert was allowed to select up to 16 of the 22 actions per case scenario, hence a maximum of 80 actions across 5 cases. The actions recommended by the algorithm for the study case scenarios were used to assess it.

We explained the survey procedure to the human experts before asking them to study the case scenarios and the accompanying set of possible actions to consider for managing each case. The expert would then recommend the most important actions for each case scenario given its conditions. The algorithm also recommended actions to the same cases based on results of an earlier study of a larger group of experts and literature. Experts in this study, however, were not aware of the algorithm nor other experts’ action recommendations. They were invited to suggest possible modifications to the actions list for clarity and to provide better decision support for the case conditions.

We analyzed data to determine the unadjusted and weighted interexpert pairwise sensitivity [18,24], false-positive rates (FPRs), and reliability coefficients. Pairwise sensitivity was calculated for each pair of experts; for instance E3–E4 is the sensitivity of E4 with respect to E3 as reference. The sensitivity of the LaD algorithm versus each human reviewer (E–LaD) was also calculated to determine how LaD–human expert scores compare with interhuman expert pairwise (E–E) scores. FPRs were calculated for the unadjusted scores. The weight assigned to an action was determined by the number of experts that selected that action for a given case scenario. That is, an action weighed 1.0 if all 5 experts selected it as important, 0.6 if 3 selected it, and 0 if none selected it. Therefore, the weights were assigned after data entry. We compared the LaD algorithm scores with averages of the human pairwise scores for each case and across all scenarios. To rank the algorithm and human experts, we compared the lower border for the 95% CI of the mean sensitivity and the upper border of its mean FPR confidence interval with corresponding values for the experts. A larger number of the lower limit border for the sensitivity confidence interval and a smaller number of the upper limit for the FPR confidence interval meant a superior rank [21].

## Results

### Overview of Case Scenarios

A total of 5 of the 6 case scenarios were managed by all experts while the sixth was completed by 2 experts. The experts articulated that the noncompleted case was similar to another they had answered and saw no big difference in general management.

As indicated in Table 2, for the 5 case scenarios together, the experts selected an average of 39 actions of a possible 80. Across the experts, case scenarios 1 and 5 received most actions with an average of 11 each, whereas case scenario 2 needed the fewest actions at 5. From the unadjusted data, unlike the experts, the LaD algorithm had most actions for case scenario 3, but there was no difference in the weighted scores.

**Table 2:** Number of actions selected per patient (actual and adjusted values).

Evaluator	P1^a^			P2			P3			P4			P5	
	Action	Adjusted value	Action		Adjusted value	Action		Adjusted value	Action		Adjusted value	Action		Adjusted value
E1^b^	9	7.2	5		3.4	5		4	6		3.4	11		8
E2	8	6.8	5		3.4	8		4.8	5		3.6	8		6
E3	12	7.4	4		2.8	6		3.4	9		5.2	13		9.4
E4	11	7.8	5		2.6	7		3.6	4		1.4	8		5
E5	13	8.8	5		3.4	5		3.6	9		4.6	14		9.6
Mean (SD)	10.6 (2.1)	7.6 (0.8)	4.8 (0.4)		3.1 (0.4)	6.2 (1.3)		3.9 (0.6)	6.6 (2.3)		3.6 (1.5)	10.8 (2.8)		7.6 (2.0)
Labor and delivery algorithm (LaD)	8	4.6	7		3.4	12		5.4	7		3.8	8		5.6

^b^P: patient case scenario.

^a^E: expert.

### Pairwise Sensitivity and FPRs for the Experts and the LaD Algorithm

**Figure 2:**
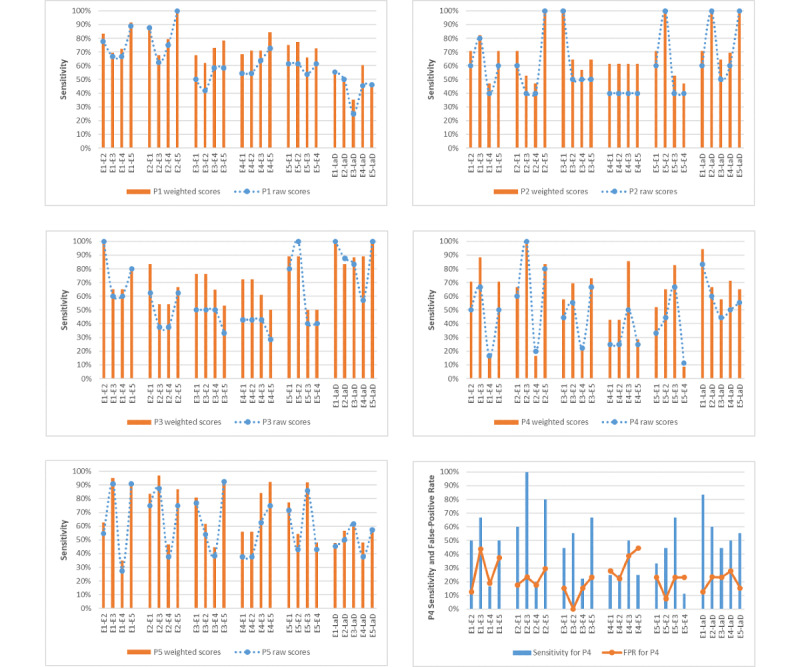
The interrater pairwise sensitivity scores for the five cases.

**Figure 3:**
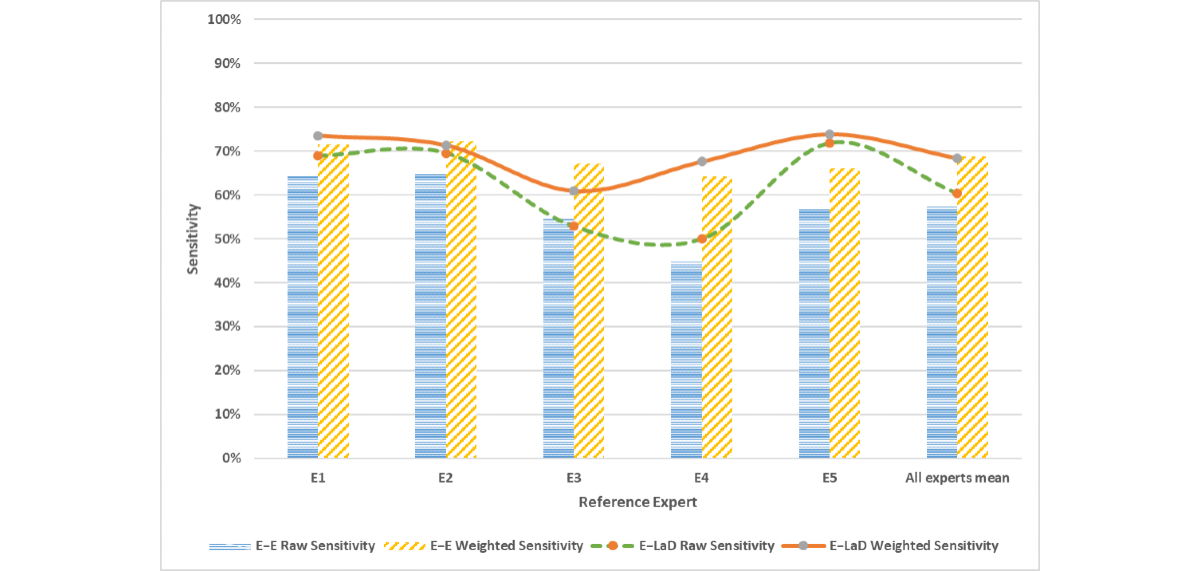
Comparison of the overall weighted and unadjusted pairwise sensitivity scores.

The interrater pairwise sensitivity for the experts and the LaD algorithm is shown in Figure 2. The mean for unadjusted pairwise sensitivity among experts (E–E) for all cases was 57.2% (SD 7.86; 95% CI 47.4-67.0), whereas the weighted mean sensitivity was 68.2% (SD 6.95; 95% CI 59.6-76.8). The difference between these means was significant (SD 11.0; 95% CI 2.8-21.2, *P*=.01). With reference to the experts, the mean sensitivity scores of the LaD algorithm (E–LaD) were 62.6% (SD 17.01; 95% CI 41.5-83.7) and 69.4% (SD 17.95; 95% CI 47.1-91.7) before and after adjustment, respectively. The difference of 6.8 in E–LaD means the 95% CI of –14.9 to 28.5 was not statistically significant, *P*=.32). As shown in Figure 3, the weighted pairwise sensitivity for experts was significantly higher (*P*=.02) and closer to the LaD sensitivity than the unadjusted scores, especially when E4 was the reference expert. The algorithm was more sensitive than E1, E4, and E5, but less sensitive than E3.

For the 5 patient cases, the average FPR of experts ranged from 12% to 33% with a mean of 23.9% (SD 9.14; 95% CI 12.6-35.2), whereas that for the E–LaD ranged from 18% to 43% with a mean of 26.3% (SD 10.43; 95% CI 13.3-39.3). Table 3 shows that case 2 was an outlier (in left tail) for the expert-to-expert pairwise false-positive scores and case 3 was an outlier (right tail) for the expert-to-algorithm FPR scores.

**Table 3:** Pairwise sensitivity and false-positive rates of experts and the labor and delivery (LaD) algorithm.

Comparisons	P1^a^	P2	P3	P4	P5	Mean (SD)	95% CI for mean	CI for difference of 2 means
E–E^b^ pairwise sensitivity: unadjusted	65.9	56.5	55.0	45.6	62.8	57.2 (7.86)	47.4 to 67.0	–11.0 to 21.8
E–LaD pairwise sensitivity: unadjusted	44.4	74.0	85.6	58.7	50.3	62.6 (17.01)	41.5 to 83.7	
E–E pairwise sensitivity: weighted	75.9	67.2	68.6	57.3	71.9	68.2 (6.95)	59.6 to 76.8	–15.7 to 18.1
E–LaD pairwise sensitivity: weighted	49.3	80.8	92.1	71.0	54.0	69.4 (17.95)	47.1 to 91.7	
E–E pairwise FPR^c^ for an action	33.1	12.2	18.3	23.2	32.9	23.9 (9.14)	12.6 to 35.2	–9.8 to 14.6
E–LaD pairwise FPR for an action	30.2	19.7	43.0	20.5	18.3	26.3 (10.43)	13.3 to 39.3	
E–E pairwise sensitivity for an action: unadjusted	65.9	56.5	55.0	45.6	62.8	57.2 (7.86)	47.4 to 67.0	2.8 to 21.2
E–E pairwise sensitivity for an action: weighted	75.9	67.2	68.6	57.3	71.9	68.2 (6.95)	59.6 to 76.8	
E–LaD pairwise agreement for an action: unadjusted	44.4	74.0	85.6	58.7	50.3	62.6 (17.01)	41.5 to 83.7	–14.9 to 28.5
E–LaD pairwise agreement for an action: weighted	49.3	80.8	92.1	71.0	54.0	69.4 (17.95)	47.1 to 91.7	

^a^P: patient case scenario.

^b^E: expert.

^c^FPR: false-positive rate.

### Determining the Rank of LaD Algorithm Among Human Experts

The 95% CIs for the mean sensitivity scores of the algorithm and the human experts showed that the LaD algorithm had a higher upper limit before and after adjustment to the mean. By contrast, the lower limit of the confidence interval for the expert FPR mean was lower than that of the interval for the LaD algorithm mean. There was a positive correlation (mean *r*^selection^ of 0.67 [SD 0.06]) in the actions that the expert pairs selected for the different patient cases (Table 4) with a reliability coefficient close to 1 (α=.91). This meant that the study experts agreed on most actions necessary for the cases and the same actions were likely to be recommended by these or other experts for the given patient scenarios.

Finally, we needed to know whether the differences in the mean sensitivity and FPR of the LaD algorithm and human experts were significant. The difference in mean sensitivity was 5.4 (95% CI –11.0 to 21.8) for the unadjusted means and 1.2 (95% CI –15.7 to 18.1, *P*=.57) for the weighted means. Because both intervals crossed the null, there was no statistical difference in the sensitivity of the experts and algorithm. In addition, the mean FPR of the experts and the algorithm was not significantly different with a 95% CI of –9.8 to 14.6 (*P*=.69).

On the basis of these sensitivity and false-positive scores, we found no statistical difference between the LaD algorithm and human experts recommending actions to childbirth monitoring health workers.

**Table 4:** Correlation and reliability coefficients of experts’ choices of actions for the cases.

Comparisons	Selection correlation coefficient of actions selected by experts for each case, *r*^selectiona^						Reliability coefficient, α^b^
	P1^c^	P2	P3	P4	P5		
E1–E2^d^	0.857	0.706	0.913	0.686	0.722		
E1–E3	0.685	0.907	0.705	0.714	0.877		
E1–E4	0.703	0.538	0.685	0.275	0.443		
E1–E5	0.829	0.706	0.848	0.607	0.844	.925	
E2–E1	0.857	0.706	0.913	0.686	0.722		
E2–E3	0.649	0.583	0.644	0.832	0.772		
E2–E4	0.751	0.538	0.625	0.267	0.511		
E2–E5	0.879	1.000	0.770	0.737	0.685	.923	
E3–E1	0.685	0.908	0.705	0.713	0.876		
E3–E2	0.648	0.583	0.644	0.832	0.772		
E3–E4	0.720	0.593	0.629	0.445	0.613		
E3–E5	0.719	0.583	0.514	0.777	0.927	.919	
E4–E1	0.703	0.538	0.760	0.275	0.443		
E4–E2	0.751	0.538	0.626	0.268	0.511		
E4–E3	0.720	0.593	0.629	0.445	0.613		
E4–E5	0.782	0.538	0.500	0.158	0.664	.861	
E5–E1	0.829	0.706	0.843	0.607	0.844		
E5–E2	0.879	1.000	0.770	0.737	0.685		
E5–E3	0.719	0.583	0.514	0.777	0.926		
E5–E4	0.783	0.538	0.500	0.158	0.664	.922	
Mean (SD)	0.757 (0.073)	0.669 (0.159)	0.687 (0.129)	0.550 (0.237)	0.706 (0.152)	.910 (0.027)	

^a^*r*^selection^ is an extension to Pearson *r* = square root of (sensitivity AB × selectivity AB), where selectivity RT = sensitivity TR. This is the selectivity for a test expert T against a reference expert R.

^b^α = kR/(1 + [k–1]R), where k is the number of experts and R is the average correlation of all expert pairs.

^c^P: patient case scenario.

^d^E: expert.

## Discussion

### Principal Findings

The search for an ideal labor and delivery monitoring decision support tool is ongoing and this study was one of many attempts to improve these tools. We have described the design of the LaD algorithm and validated it through comparison of its logic with human experts of childbirth monitoring. We found the algorithm to be equivalent in sensitivity and FPRs to experts with high reliability, that is, its action recommendations were close to the clinically “correct” ones. In clinical situations, lack of a gold standard against which to evaluate tools meant that traditional device validation tests were inappropriate and so childbirth experts had to act as the reference silver standard as in most types of clinical decision making [20,24]. Like Scheuer et al [21], we used the selection correlation coefficient *r*^selection^ (an extension to Pearson *r*) because clinical experts often agree on many nonimportant actions for any patient case [18]. Most childbirth actions are not selected independent of one another, so our results would be less trustworthy if we used the kappa or pi statistics for measuring agreement. Likewise, we could not use Gwet AC statistic that necessitated assigning constant weights based on gold standards to parameters for all the patients, which would not be rational in our scenario [18,25]. The results of this study can be used to develop an abridged and more appropriate paper- or computer-based labor monitoring decision support tool that is less contentious than the WHO-modified partograph.

### Limitations

The main limitations to this study are as follows: First, the low number of patient cases rated by the experts. Patient clinical scenarios have subtle or major differences that it would be virtually impossible to expect an exhaustive tool or validation. The cases were few, but each contained 22 actions to be considered; thus, the experts were not assessed on one case/condition per se, but on a sum of actions for each case and then the average of the 5. Therefore, the experts and algorithm were assessed on 110 instances summarized into 5 cases. This approach was similar to that used by Scheuer et al [21] who had over 5000 spike detections presented in under 40 scenarios [21]. Second, a total of 5 experts were not enough to tease out the effect of fast or slow actors when deciding to intervene in a clinical maternity setting. The fast actors tend to intervene too soon and so too much, whereas the slow actors intervene too late and so too late for good clinical outcomes, as expressed by Miller et al [26]. Third, the algorithm was based on suggestions from providers in low-income settings which are generally on the “too little, too late” side, and hence we expected the participants (E1, E4, and E5) to be more sensitive and E3 to be slower at acting. The strength of the pairwise sensitivity and the modified correlation we used is dampening the individual effect/biases of participants such that we still found no statistical differences between the group and the algorithm. Another limitation could have been our set of candidate actions from which experts selected. As was done by other researchers [18], we provided experts with candidate actions (from other studies) to encourage them to concentrate on relevant actions, but it could have hindered participants with divergent opinions from choosing their preferred actions. Following years of promoting the WHO partograph, some childbirth experts have got so engrained in it that any changes to its parameters could seem unfounded and unacceptable [27-29]. With these limitations in mind, we agreed that our validation results were preliminary and more assessments of the LaD algorithm would be done after its deployment and testing under more conditions.

### Conclusions

The LaD algorithm was more sensitive but with a higher FPR than the childbirth experts, although the differences were not statistically significant. An electronic tool for childbirth monitoring with fewer parameters than those in the modified WHO partograph may not be inferior to human experts in labor and delivery clinical decision support.

## Abbreviations

FPR: false-positive rate
WHO: World Health Organization
